# An Update on Medication Use in Older Adults: a Narrative Review

**DOI:** 10.1007/s40471-021-00274-5

**Published:** 2021-07-20

**Authors:** Heather E. Barry, Carmel M. Hughes

**Affiliations:** Primary Care Research Group, School of Pharmacy, Queen’s University Belfast, Medical Biology Centre, 97 Lisburn Road, Belfast, BT9 7BL UK

**Keywords:** Aging, Medications, Medication adherence, Older adults, Outcome measures, Polypharmacy, Prescribing

## Abstract

**Purpose of Review:**

The global phenomenon of population aging is impacting the health and care needs of society. The use of medications by older adults is acknowledged to be the most common form of medical intervention for many acute and chronic conditions and prescribing in this population continues to increase. In this narrative review, we summarise the age-related factors that should be considered when prescribing for older adults, address some of the perennial challenges related to medicine use in older people, and highlight important emerging research in this area.

**Recent Findings:**

A range of age-related factors should be considered when prescribing for older adults. However, the evidence base still lacks data pertaining to older adults due to their continued under-representation in clinical trials. Multimorbidity, polypharmacy, and inappropriate prescribing continue to remain prevalent among older adults, although recent research has been focused on the development and evaluation of complex interventions to address these challenges.

**Summary:**

Further high-quality studies of interventions to improve and support medication use in older adults are needed, ensuring that older adults are well represented in such trials and consideration is given to the measurement of patient- and provider-focused outcomes.

## Introduction

Population aging is occurring globally; the number of people aged 60 years and over has more than doubled since 1980 and it is estimated that this figure will double again by 2050 [1]. Whilst this demographic shift is representative of advances in medicine, technology, and public health, it also poses significant and widespread challenges to society [2]. One aspect of this is the changing burden of disease, with an overall shift from communicable diseases to chronic, non-communicable diseases [3, 4]. Whilst chronic conditions such as heart disease, stroke, chronic obstructive pulmonary disease, and cancer are acknowledged to be leading contributors to global deaths and disability [5], other conditions which are increasing in prevalence such as dementia, diabetes, and Parkinson’s disease are projected to have a greater effect on death and disability over the coming years [6–9].

Multimorbidity, defined as the presence of two or more chronic conditions—both diseases and geriatric syndromes such as frailty [10]—is a mounting global public health challenge as our population ages [11, 12] and is associated with poorer health outcomes, reduced quality of life, and increased healthcare costs [13]. With the resultant increasing complexity of managing multimorbid older adults, comes the consequence of greater treatment (specifically medication) burden. Medication usage and polypharmacy (the concurrent use of multiple medications) have increased markedly over the last number of decades [14, 15••]. The use of potentially inappropriate medications (PIMs), where potential harms outweigh potential benefits, is also prevalent among older people and associated with higher rates of healthcare utilisation and costs [16••, 17]. As a result, these complicated and intertwined issues of multimorbidity, polypharmacy, and prescribing appropriateness in older adults have been the focus of much research activity in recent years and the evidence-base is rapidly expanding.

Therefore, in this paper, we aim to summarise the age-related factors that should be considered when prescribing for older adults, address some of the key and persistent challenges relating to medicine use in older people such as polypharmacy and appropriateness of prescribing, highlight important emerging research in this area relating to intervention development and evaluation as well as the increasing importance of defining core outcome sets (COSs) for trials within this research field, and provide recommendations for future research.

## Search Methods

A literature search was conducted for relevant literature published since 2016 (i.e. last 5 years) using PubMed, Web of Science, and the Cochrane Library of Systematic Reviews. The following search terms were used: ‘older adults’, ‘older people’, ‘elderly’, ‘aged’, ‘medicines’, ‘medications’, ‘prescribing’, ‘polypharmacy’, ‘multimorbidity’, ‘primary care’, and combinations thereof. The authors’ existing knowledge of literature was also used to identify relevant references, and we hand-searched reference lists of identified articles to identify additional papers. Only peer-reviewed articles published in English were selected. Our focus for this review was papers focusing on the primary care setting with community-dwelling older adults or those residents in care homes; we therefore excluded studies of older adults in secondary care or hospice settings as this was considered outside the scope of the current review.

## Age-Related Prescribing Considerations

As a person ages, there are many normal physiological changes that take place which may affect the way in which a person processes and responds to medications. For example, reductions in gastric motility, lean body mass and body water, blood flow, and renal and hepatic function can all affect drug pharmacokinetics (absorption, distribution, metabolism, and excretion) [18–20]. Age-related pharmacodynamic changes may also occur which can increase an older person’s sensitivity to several drug classes, such as anticholinergics, benzodiazepines, and opioids [19, 21]. Adverse effects associated with such drugs may therefore be potentiated. Changes in pharmacokinetics and pharmacodynamics may be amplified in older adults with frailty, making them particularly vulnerable to adverse drug events [22, 23]. Whilst beyond the scope of this paper, a recent review has considered medication use in frailty in more detail [24].

Additional age-related changes should also be considered during the prescribing process to ensure optimal medication safety and reduce medication-related harm. Sensory impairment, particularly vision and hearing loss, and issues with swallowing, manual dexterity, and co-ordination may affect a person’s ability to acquire, administer, manage, and adhere to their medication regimen [19, 25–27]. Declining cognitive function and/or the presence of intellectual disability in older adults may also present challenges during prescribing if patients are unable to participate in shared decision-making and communicate their beliefs and preferences, as well as affecting medication management abilities [19, 28, 29]. Prescribers should be cognisant of older adults’ use of non-prescription medications, particularly given the fact that they have been shown to be high users of over-the-counter (OTC) and complementary and alternative (CAM) medicines [30–33], placing them at greater risk of potential medication-related harm through drug interactions [34, 35]. It is apparent that prescribing for older adults is not free from ambiguity or uncertainty when taking these factors into account during the prescribing process. Prescribers may be guided by accessing an appropriate evidence base; however, this is somewhat limited by the fact that such treatment guidelines often contain insufficient clinical trial data relating to older adults.

## Inclusion of Older Adults in Clinical Studies

It is acknowledged that older adults are excluded or under-represented in clinical trials [36•, 37–40]. Although age representation in influential cardiology clinical trials has been reported to have increased modestly over the last two decades, gaps in the representation of older adults still exist [41, 42]. A recent study which examined the representation of older adults in phase III clinical trials funded by the National Institutes of Health (NIH) found that one-third of studies had arbitrary upper age thresholds [36•]. However, beyond age, older adults are found to have often been implicitly excluded based on polypharmacy or comorbid conditions [36•, 43, 44•]. A review conducted by He and colleagues which examined the percentage of clinical populations with a physical health condition who would be excluded by randomised controlled trials for that condition concluded that trial evidence is typically derived from narrow populations selected to have a lower risk of adverse effects, by selective exclusion of patients with co-morbidity, co-prescribing, and frailty [44•]. Clearly, this represents a major paradox as the older population uses the most medication due to multimorbidity and are most frequently exposed to adverse drug reactions; therefore, data from such trials are not as relevant to more complex patients. Furthermore, the outcomes from trials which are under-representative of older people may not be generalisable to the older population [45]; the importance of outcomes is discussed later in this review. Over the past year, it has become evident that older adults are even under-represented in COVID-19 trials, despite being one of the populations most affected by and at risk from COVID-19 infection [46–48]. Studies have taken a siloed approach as to how co-morbidities and age affect outcomes from COVID-19 infection [46, 49], and there have been calls for COVID-19 research to be more inclusive of older people with frailty, cognitive impairment, and multimorbidity [46, 50].

There have been promising changes in trial inclusion, some of which have been mandated by government and/or regulatory bodies. For example, the NIH implemented a new policy in 2019, which mandates that all NIH-funded clinical studies must include people across the lifespan [51], and the Food and Drug Administration has issued draft guidance for inclusion of older adults in cancer clinical trials [52]. Whilst challenges remain to ensure that changes in such inclusivity practices take effect, particularly in cardiovascular research [53, 54], some improvements in the recruitment of older adults have been observed in cancer clinical trials [55] which will allow evidence to emerge in these fields.

## Prescribing in the Face of Multimorbidity

The evidence base has informed guidelines which traditionally have been focused on single conditions which, of course, can lead to polypharmacy. Despite many studies focusing on various aspects concerned with polypharmacy, one recurring problem is the difficulty and variation in the way in which it has been defined to date [56–58]. A review by Sirois and colleagues reported that more than 46 definitions of polypharmacy were retrieved from the literature [58]. Clearly, this makes exploring the prevalence of polypharmacy difficult and is one reason why such a range in prevalence has been reported [59•, 60••, 61–63].

Over recent years, the concept of appropriate versus inappropriate polypharmacy has been suggested as a more meaningful classification rather than using numerical thresholds [56, 64]. Appropriate polypharmacy involves the prescribing of appropriate combinations of medications to people with multimorbidity, whilst inappropriate polypharmacy can give rise to potentially inappropriate prescribing and prescribing of potentially inappropriate medications [56, 57, 64]. Psychotropic polypharmacy remains problematic [65], and polypharmacy can be associated with certain classes of medications, particularly central nervous system (CNS) drugs, anticholinergics, sedatives, and proton pump inhibitors [66–68]. Several epidemiological studies have demonstrated that potentially inappropriate prescribing increases with polypharmacy [69–73]. Similar trends have been observed in care home populations and people with dementia [74, 75]. Prescribing cascades, where an adverse drug event is not recognised as such and an additional drug is prescribed to treat the drug-induced adverse event, may contribute to polypharmacy and create additional risks for multimorbid patients [76, 77]. McCarthy and colleagues have highlighted that appropriate and therapeutically beneficial prescribing cascades can also exist; analogous to the way that polypharmacy can be classified as appropriate or inappropriate, prescribing cascades may be appropriate or problematic [77]. ‘Legacy prescribing’ (drugs that should be prescribed for an intermediate period of time, longer than three months but not indefinitely, that are not appropriately discontinued) has also been identified as a contributor to polypharmacy, with antidepressants, bisphosphonates, and proton pump inhibitors commonly implicated [78]. Von Buedingen and colleagues have cautioned against cross-sectional assessments of medication use due to the frequent changes they observed in the medication regimens of older adults with multimorbidity and polypharmacy [79]. These are important considerations when interpreting epidemiological studies of polypharmacy and prescribing appropriateness, and the goal of achieving patient-centred care should not be forgotten. An international group of academics and clinicians have set out ten recommendations for action for reducing inappropriate medication use and polypharmacy; giving priority to patient and family preferences is emphasised [80].

Unsurprisingly, as the number of drugs prescribed to older adults increases, so too do issues with adherence. Increasing age is not necessarily a predictor of non-adherence, although adherence is more likely to be lowest in older age categories of older adults [81, 82]. There is greater recognition that adherence is a complex health behaviour with multifaceted determinants [83], and as a result, careful thought is needed when planning how to resolve barriers to adherence. For example, a recent study reported that cost-related medication non-adherence is becoming increasingly common among older adults in the USA [84]; however, there are myriad other reasons for medication non-adherence including patient-related, socioeconomic, and therapy-related factors [85]. This has important consequences for intervention developers who must take a broad and holistic approach to solving all medication-related challenges, not just those related to adherence.

## Interventions to Support Medication Use in Older Adults

There is an increasing focus on developing, evaluating, and implementing interventions to support prescribing and medication use in older people. Over the last number of years, Cochrane reviews have been published on interventions to optimise prescribing for older people in care homes [86], improve the appropriate use of polypharmacy in older people [87], and improve medication-taking ability and adherence in older adults prescribed multiple medications [88]. Although each of these reviews was focused on different aspects of medication use in older adults, it is interesting to note that all concluded that there was great variability between the interventions that were included, making it difficult to draw firm conclusions. The need for high-quality studies, measuring well-defined and relevant outcomes has been emphasised.

It is evident from the literature that the development of complex interventions is increasingly following the United Kingdom’s Medical Research Council guidance by taking a systematic approach underpinned by the evidence base and relevant theory and supported by thorough piloting and feasibility work [89]. Indeed, Patton and colleagues highlighted the need for a more robust theoretical basis for interventions targeting medication adherence in older adults [90]. A number of interventions to support medication use in older adults have been developed in this systematic manner [91–93]. The supporting prescribing in older patients with significant multimorbidity and polypharmacy (SPPiRE) intervention is designed to support general practitioners to reduce potentially inappropriate prescribing and consider deprescribing through an individualised structured medication review in older people with multimorbidity and polypharmacy in primary care in the Republic of Ireland [91]; the definitive RCT is ongoing. The Solutions for Medication Adherence Problems (S-MAP) intervention is theory-based and guides community pharmacists using a web application to identify medication adherence barriers and deliver individually tailored solutions (behaviour change techniques) [92]. It has undergone pilot testing in community pharmacies in Northern Ireland and England; whilst study procedures and intervention delivery were reported to be feasible, the authors plan to make modifications before progressing to a cluster RCT to explore intervention effectiveness [92]. Other approaches that have been utilised in interventions to support prescribing and medication use in older people include electronic clinical decision support systems [94, 95] and deprescribing [96, 97]. Whilst deprescribing interventions may reduce mortality and the use of potentially inappropriate medications, and improve medication adherence [96, 98, 99], the effect may not be sustained, and further research is needed to determine the optimal interval of repeated deprescribing interventions [97]. A multi-faceted intervention for primary care has been proposed that includes an adapted version of a deprescribing protocol which may reverse prescribing cascades [100]. Novel approaches continue to be discussed within the literature. For example, a recent study has identified a core set of 12 indicators of clinical importance considered relevant to polypharmacy appropriateness which could be used to target and monitor future polypharmacy interventions [101]. It has been suggested that pharmacogenomics might help in individualised deprescribing in older adults [102], whilst machine learning and big data analysis techniques have been used to predict, identify, and manage polypharmacy [103, 104]. It will be interesting to see how these areas may help to progress the field over the coming decades.

There is consensus in the literature that many published trials of interventions to support prescribing and medication use in older adults are heterogeneous with respect to the outcomes measured across studies or selectively report outcomes, making it difficult to conclude which interventions are most effective [105, 106]. As a result, over recent years much work has focused on the development of Core Outcome Sets (COSs) to be used in trials focusing on optimising prescribing in care homes [107], medication review [108], multimorbidity [109], improving appropriate polypharmacy [110], and medicines management in people with dementia [111]. Aubert and colleagues have argued that studies of interventions to reduce inappropriate medications should include outcome measures that are more clinically meaningful and centred on both patients and healthcare providers [112]. Patient-centred outcomes research should be used to generate evidence to facilitate person-centred care, the importance of which has been increasingly recognised and forms a central tenet to many evidence-based clinical guidelines. It is important, therefore, that patients, carers, and healthcare professionals are included during the COS development process so that the final COS has relevance to all key stakeholders. Further work is needed to reach consensus-derived agreement on the selection of outcome measurement instruments in previously developed COSs.

## Conclusion

This review examined some of the key pertinent issues facing older adults in relation to prescribing and the use of medicines. Multimorbidity, polypharmacy, and potentially inappropriate prescribing remain major challenges for healthcare providers, and future research must continue to focus on developing and evaluating novel interventions to address these challenges, with the recruitment of older people into the studies to assess effectiveness. Higher-quality studies of such interventions are needed, with a focus on measuring outcomes of clinical importance to key stakeholders.

## Data Availability

Not applicable.
